# James Chau: connecting people with public health

**DOI:** 10.2471/BLT.19.030819

**Published:** 2019-08-01

## Abstract

James Chau talks to Ana Lesher Treviño about the challenges faced in communicating about public health.

Q: How did you become interested in public health?

A: I covered the SARS epidemic in 2003 as a correspondent in Hong Kong. So I suppose that was the beginning of my professional connection. On the personal side, I have struggled with depression for many years and have had several experiences that make me acutely aware of health issues, particularly the stigma associated with certain illnesses. HIV is an obvious example. A friend’s brother recently died from an AIDS-related illness. One evening, he shared his story and his grief. I was profoundly moved by the incredible trauma his family was experiencing: losing a son and brother they loved so much, and yet grieving his death at a distance because of the stigma and shame some of them were feeling.

Q: You were appointed Goodwill Ambassador for UNAIDS in 2009 and for WHO in 2016. How do you see your role?

A: I see myself as someone who is tasked to communicate and facilitate, someone who can merge ideas, interests and solutions for the good of public health. I am deeply honoured to be working alongside people from WHO. Their decisions impact us all and I've seen how the policy they create is transforming populations. To be a very small cog in that enormous and respected wheel is very exciting, partly because I can apply the lessons I learned in working on HIV with UNAIDS across a wider range of health issues. The AIDS epidemic and the communications initiatives developed in response to it offer many valuable lessons: how to deal with the scientific aspects, how to communicate and interact with people, civil society and political leadership. These lessons have fundamental universal applications: the importance of putting scientific evidence at the heart of a response, for example, and the importance of following through with a genuinely inclusive communications process that reflects different points of view.

Q: What are the main challenges in communicating around the sustainable development goals (SDGs)?

A: The 17 goals and the 169 targets constitute a tremendous project, a kind of ‘moon shot’ for a more fulfilled humanity. However, because there are so many goals and targets, they can be hard for many people to grasp. This presents a challenge to their visibility and buy-in from the communities that need to be fully engaged to advance the SDG agenda.

“To achieve a genuine social movement, you need to reach people.”

Q: Do you think people are unaware of the SDGs?

A: If you went out on the street, and if that street was not next to the Palais des Nations in Geneva, you would meet a lot of people who wouldn’t know what the three letters, SDG, stand for. And that’s stunning because more than 190 countries have signed off on this global agenda. The success of the SDGs depends not only on the leadership of heads of state and government – communities must also be allowed to own the SDGs and to drive the different initiatives that are required to realize them. It doesn’t help that the acronym is opaque. If we said, ‘Global Goals’ it would be easier for people to understand how ambitious it is, but also how comprehensive. One of the biggest challenges is getting this idea across in a way that people can understand and can also relate to. Many years before he became WHO Chef de Cabinet, Bernhard Schwartländer would tell me that to achieve a genuine social movement, you need to reach people – and to do that, you need to connect as one human being to another.

Q: Speaking of goals and targets, how do you think we should be communicating on progress?

A: It comes down to ensuring transparency and accountability. Commitments have been made and I think it is important to keep citizens informed about where we are in fulfilling them. We need independent review, we need civil society and academia involved, and we need a mechanism for redress when commitments aren’t kept. Without these, calls for accountability will always ring hollow. So, we shouldn’t be afraid to ask “why not?” the next time our leaders fail to deliver.

Q: What do you consider the biggest challenges in communicating about public health generally?

A: To begin with, there is the complexity of the issues involved. Public health can be overwhelming for people who are not trained in public health. So, it’s important to ensure that the people on the frontlines of public health are provided with the support and guidance they need to communicate as clearly as possible without oversimplifying. WHO is the world’s leading health agency and an important part of what it does is highly technical. It’s filled with experts who have been trained to a very high level in what they do. We need to leverage their skills, talent and expertise outside of their immediate areas, so that the important work they do enriches the knowledge of the mainstream populations they serve.

Q: Should we train scientists themselves to write in a more accessible way?

A: That’s an interesting idea: in my experience, some scientists are willing to do this and others are not. Leading international experts have often committed decades of their life to one discipline and may not feel that communication should be added to that workload.

Q: So, the journalists need to do a better job?

A: They need to make sure that whatever they are writing or talking about is grounded in the evidence, in the facts. And not just journalists, of course. I’m really talking about anyone working in communications.

Q: Does that mean that journalists need to become experts in the topics they write about?

A: That’s probably too much to ask, but they need to have a good grasp of the topics they write about based on their own research and their interactions with the experts. There are advantages to remaining outside the technical discourse to a certain extent. Being an outsider gives me an advantage in my work with WHO because I am positioned between different constituencies. Also, it is vital that journalists speak to the heart and that they ground their writing in compelling human stories and experience. It is that mix of the technical and the human in WHO that makes it so exciting. I have always seen it as a technical agency with a humanitarian mission that must absolutely connect with the Member States and the communities it serves at both the technical and human level. Finally, I believe that journalists and communicators need to think very carefully about the impact their messages and stories can have, and craft their content accordingly. The media have at times dropped the ball in terms of responsible reporting.

“It is that mix of the technical and the human in WHO that makes it so exciting.”

Q: Can you give an example?

A: For example, you can make the argument that the media have painted an overly optimistic picture regarding HIV/AIDs and that this in turn has created a perception, especially among young people, that AIDS was a problem for their parents’ generation, and not for them. Of course, the global health community and the communicators for that community have their part of responsibility in this also.

Q: Can you talk about that a little?

A: Putting it simply – and I am thinking about my own experience with UNAIDS here – because we wanted to address stigma and discrimination, we pushed a message saying that people living with HIV have a shot at living a very fulfilling life, physically, emotionally and sexually. Of course, this reflected the reality that more effective treatments were appearing and becoming more accessible. But, as a result of that messaging, a certain complacency has crept in, especially among young people, while the news media have moved on to other issues to fill their headlines of the day, be it terrorism, climate change or refugees. AIDS has not gone away and neither have my friends who live with HIV. So, we had good intentions in the way we framed that issue, but I worry very much about the impact of this and with hindsight, I feel that the communication strategy could have been more thoughtful at the time – more thoughtful and careful. That is a lesson we can now apply to other health challenges.

Q: Such as vaccination, for example?

A: Yes. We need to strike the right balance between communicating the need for vaccines and immunization, while also taking on board real concerns around safety. We need to remind people that vaccinating your child is not a lifestyle choice, but a social responsibility. This returns me to what I was saying about the importance of journalism that is based on the evidence, based on the facts. And we need to insist on those facts being heard. This will require maximum collaboration between journalists and scientists committed to getting the truth out there to counter the fake news that we are bombarded with every day, particularly through social media. Because we have to acknowledge the fact that while social media and digital media have provided exciting new opportunities, they have also given rise to significant challenges, one being the emergence of influencers who have no scientific background, giving advice on public health issues. And while we are discussing vaccines, may I put this on the record? Vaccines are among the best, most effective and safest interventions that we have to keep people healthy, especially children. 

Q: Is presenting the evidence in clear and compelling ways sufficient to push back against false information and fake news?

A: I don't think any of us has a whole answer to that question. But there are certain constituencies of people, who will write what they want to write and believe what they want to believe. Unfortunately, we are limited in our response. But we can push a lot harder to present the facts and to call out fake news when we see it. It is complex – but so is the world and the safer, healthier and happier global future we are working towards. That’s the beautiful opportunity.

